# Guiding Myocardial Revascularization by Algorithmic Interpretation of FFR Pullback Curves: A Proof of Concept Study

**DOI:** 10.3389/fcvm.2021.623841

**Published:** 2021-03-11

**Authors:** Jean-François Argacha, Jean Decamp, Bert Vandeloo, Danilo Babin, Stijn Lochy, Karen Van den Bussche, Quentin de Hemptinne, Panagiotis Xaplanteris, Julien Magne, Patrick Segers, Bernard Cosyns

**Affiliations:** ^1^Department of Cardiology, Universitair Ziekenhuis Brussel, Vrije Universiteit Brussel (VUB), Brussels, Belgium; ^2^Centre for Quantum Technologies, National University of Singapore, Singapore, Singapore; ^3^imec-TELIN-IPI, Ghent University, Ghent, Belgium; ^4^Department of Cardiology, Centre Hospitalier Universitaire (CHU) Saint-Pierre, Université Libre de Bruxelles (ULB), Brussels, Belgium; ^5^Department of Cardiology, Dupuytren University Hospital 2, Limoges, France; ^6^INSERM U1094 and IRD, Limoges University, Limoges, France; ^7^Department of Electronics and Information Systems, IBiTech-bioMMeda, Ghent University, Ghent, Belgium

**Keywords:** coronary artery disease, percutaneous coronary intervention, pullback, computer science, virtual coronary stenting, vessel disease distribution, fractional flow reserve, coronary physiology

## Abstract

**Background:** Coronary artery disease distribution along the vessel is a main determinant of FFR improvement after PCI. Identifying focal from diffuse disease from visual inspections of coronary angiogram (CA) and FFR pullback (FFR-PB) are operator-dependent. Computer science may standardize interpretations of such curves.

**Methods:** A virtual stenting algorithm (VSA) was developed to perform an automated FFR-PB curve analysis. A survey analysis of the evaluations of 39 vessels with intermediate disease on CA and a distal FFR <0.8, rated by 5 interventional cardiologists, was performed. Vessel disease distribution and PCI strategy were successively rated based on CA and distal FFR (CA); CA and FFR-PB curve (CA/FFR-PB); and CA and VSA (CA/VSA). Inter-rater reliability was assessed using Fleiss kappa and an agreement analysis of CA/VSA rating with both algorithmic and human evaluation (operator) was performed. We hypothesize that VSA would increase rater agreement in interpretation of epicardial disease distribution and subsequent evaluation of PCI eligibility.

**Results:** Inter-rater reliability in vessel disease assessment by CA, CA/FFR-PB, and CA/VSA were respectively, 0.32 (95% CI: 0.17–0.47), 0.38 (95% CI: 0.23–0.53), and 0.4 (95% CI: 0.25–0.55). The raters' overall agreement in vessel disease distribution and PCI eligibility was higher with the VSA than with the operator (respectively, 67 vs. 42%, and 80 vs. 70%, both *p* < 0.05). Compared to CA/FFR-PB, CA/VSA induced more reclassification toward a focal disease (92 vs. 56.2%, *p* < 0.01) with a trend toward more reclassification as eligible for PCI (70.6 vs. 33%, *p* = 0.06). Change in PCI strategy did not differ between CA/FFR-PB and CA/VSA (23.6 vs. 28.5%, *p* = 0.38).

**Conclusions:** VSA is a new program to facilitate and standardize the FFR pullback curves analysis. When expert reviewers integrate VSA data, their assessments are less variable which might help to standardize PCI eligibility and strategy evaluations.

**Clinical Trial Registration:** https://www.clinicaltrials.gov/ct2/show/NCT03824600.

## Introduction

Percutaneous coronary intervention (PCI) in chronic coronary syndrome remains a controversial issue as several studies failed to show benefit on cardiovascular mortality (1) or symptom improvement (2). As the potential advantage of revascularisation might depend on the severity and the extent of ischemia in chronic syndromes, international guidelines have recommended the measurement of the fractional flow reserve (FFR) to improve the selection of patients and vessels suitable for myocardial revascularization (3). Compared to medical treatment, FFR-guided PCI improves symptomatic relief and reduces a composite endpoint consisting of death, myocardial infarction, and urgent revascularization at 5 years (4). A meta-analysis recently confirmed that FFR-guided PCI reduced the composite endpoint of cardiac death or myocardial infarction compared with medical therapy (5).

In addition to patient selection according to baseline FFR, FFR improvement after PCI is also an independent predictor of future major adverse cardiovascular events (6). At the same time, still approximately one-third of patients have a sub-optimal physiologic result after FFR guided PCI (7). Various invasive strategies have been developed to maximize the post-PCI FFR gain. Most of these strategies rely on the evaluation of the pattern of vessel disease through a pullback evaluation of the hyperemic (FFR) or non-hyperemic pressure ratios (e.g., instantaneous-wave free ratio [iFR]). The analysis of pressure pullback curves reveals that the residual resistance outside the stented lesion, typically observed in diffuse diseases, is one of the mechanisms related to sub-optimal improvement of post-PCI FFR (8). Moreover, the assessment of disease distribution has to be taken into account, as stent implantation is more efficient to remove focal than diffuse coronary resistance (9). Thus, suboptimal FFR after PCI may be improved by the evaluation of epicardial resistance distribution. However, the interpretation of FFR pullback (FFR-PB) curves by visual inspection remains complex and subjective. Indeed, two levels of expertise are required for the interpretation of coronary pressure-wire pullback data: firstly, the operator needs to determine the hemodynamic eligibility of a PCI throughout the evaluation of the vessel disease pattern and secondly, the operator needs to define a PCI strategy integrating both coronary physiology and anatomy. Although several studies proposed focal (abrupt) pattern and diffuse (gradual) patterns of FFR pullback curves, there are no clear criteria to define the physiological patterns of disease. Recently, the pressure pullback gradient (PPG) was proposed to characterize the functional pattern of coronary artery disease (10). This index analyzes the maximal variation in FFR signal over a length of 20 mm. A PPG index value close to zero suggests a diffuse distributed disease, whereas a value close to one suggests more focally distributed disease. Adding computer sciences for the interpretation of pullback curves, integrating all the FFR variations occurring along the vessel, might further improve treatment decision-making throughout clear-cut diagnostic features and PCI recommendations. Therefore, this study proposes a virtual stenting algorithm (VSA) which aims to provide a vessel disease characterization by quantifying the longitudinal variation of the FFR signal within this vessel, and a detection of high-pressure loss which might be amenable to a stent placement. Computing the FFR-PB curves opens some new possibilities to define the optimal PCI strategy which will most adequately eliminate the majority of pressure loss across the vessel.

As the VSA is a new computer science concept, its clinical relevancy was tested in a survey analysis of the rating of 39 vessels by five cardiologists. We hypothesize that VSA would increase rater agreement in interpretation of epicardial disease distribution and subsequent evaluation of PCI eligibility.

## Methods

### Study Design

**Figure 1:**
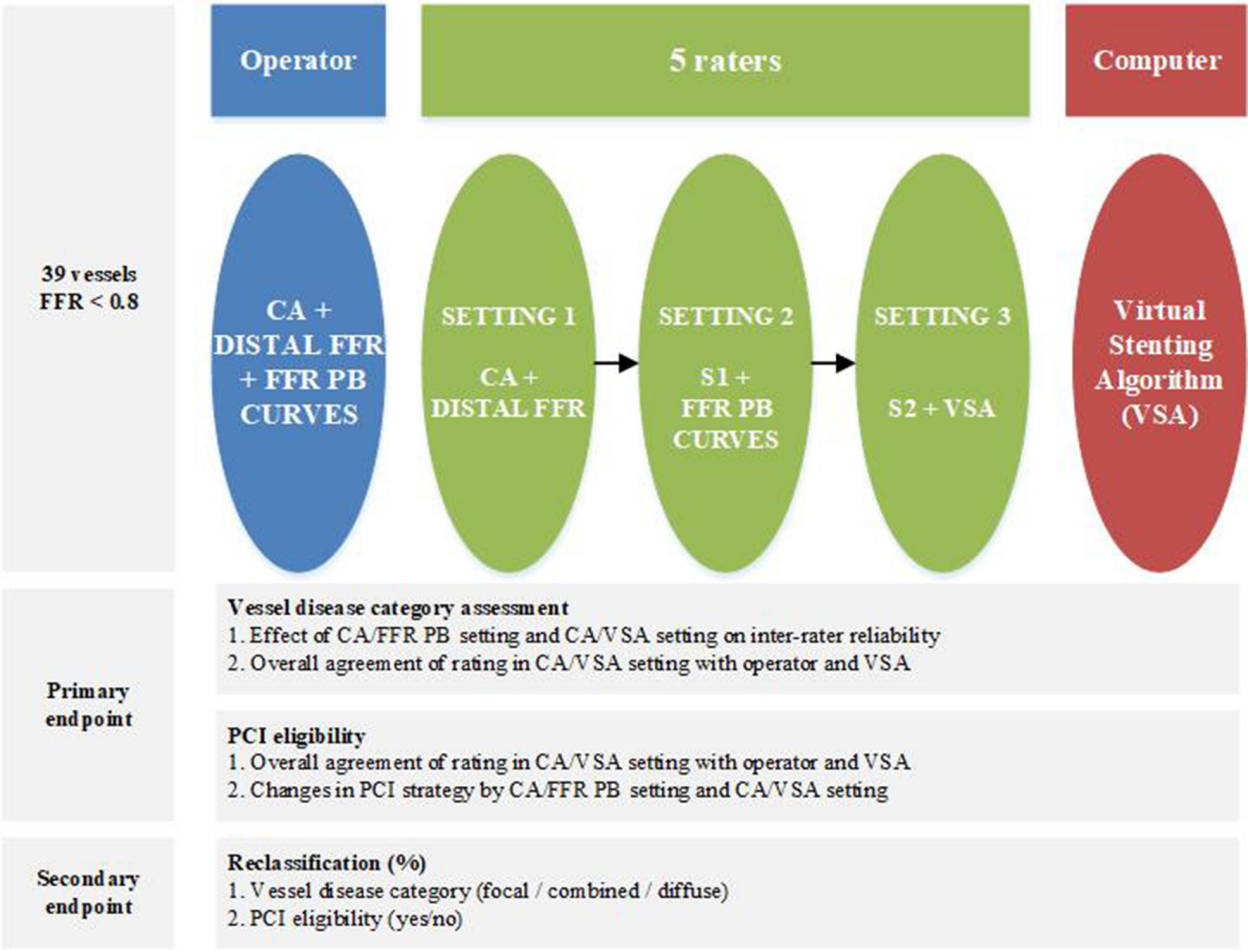
Study design.

From a prospective FFR-PB registry of patient suffering of chronic coronary syndrome, all vessels with a 30–90% diameter stenosis on visual inspection of CA and a distal FFR of <0.8 were included. Exclusion criteria to participate to this registry were acute coronary syndromes, previous coronary artery interventions, significant valvular disease, severe obstructive pulmonary disease or asthma bronchial and severe tortuosity or severe calcification. Six experienced interventional cardiologists, participated in this study. An operator, who actually performed the case evaluated epicardial disease distribution and PCI eligibility of the vessel based on coronary angiography and FFR curves. The five remaining interventional cardiologists served as raters and performed their evaluation independently and blinded to each other in three consecutive settings: first, based on CA and distal FFR (CA/FFRdist); second, based on CA and FFR-PB curve (CA/FFR-PB); and finally, based on CA and VSA (CA/VSA) (Figure 1).

Epicardial disease distribution was classified as focal, combined or diffuse by the operator and the different raters in each consecutive setting. This classification was done at the discretion of each cardiologist based on their subjective interpretation of the pattern of narrowing of the luminal silhouette on coronary angiography and, in the second setting, by integrating also the pattern of the FFR pullback curve. Indeed, there are no widely accepted angiographic or physiologic definitions of what is considered as a focally or a diffusely distributed coronary disease. Finally this assessment was assisted by the VSA. In every setting, each cardiologist was asked to report the vessel eligibility for PCI treatment, and if eligible, the PCI strategy. PCI eligibility was defined as a binary decision that the vessel is eligible, or not, for a stent placement to restore coronary blood flow. PCI strategy was defined as the stent position, the stent length and the number of stents that would be required to ensure adequate restoration of coronary blood flow.

The primary endpoint of this study was to assess the effect of introducing a VSA analysis of FFR-PB curves on the inter-rater agreement in the classification of distribution of epicardial resistance, PCI eligibility, and on the changes in PCI strategy. The secondary endpoint was to assess the reclassification of vessel disease category and the related changes in PCI eligibility at each consecutive setting.

### Distal FFR and FFR-PB Measures

Distal FFR measurements were performed according to standard recommendations (11), and during a continuous intravenous adenosine infusion at a dose of 140 μg/kg/min with a pressure wire (PressureWire X, St Jude Medical, Minneapolis, USA) in the distal vessel. After obtaining steady-state hyperaemia to ensure the measurement of distal FFR, a pressure wire pullback was performed at a speed of 1 mm/s using a motorized system (Volcano R 100, San Diego CA, USA) adapted to grip the pressure wire under continuous pressure recording. The reproducibility analysis of the FFR-PB using this motorized system has been previously reported (12). One FFR value per 0.01 mm was recorded and used to generate the FFR-PB curve. FFR-PB pressure recordings were extracted from the RadiAnalyzer Xpress (St Jude Medical, Minneapolis, USA) or the QUANTIEN Integrated FFR System (Abbott Vascular, Illinois, USA) and were then offline imported into the VSA. Post-PCI FFR measurements were performed in a subgroup of vessels at the discretion of the operator.

### Virtual Stenting Algorithm

**Figure 2:**
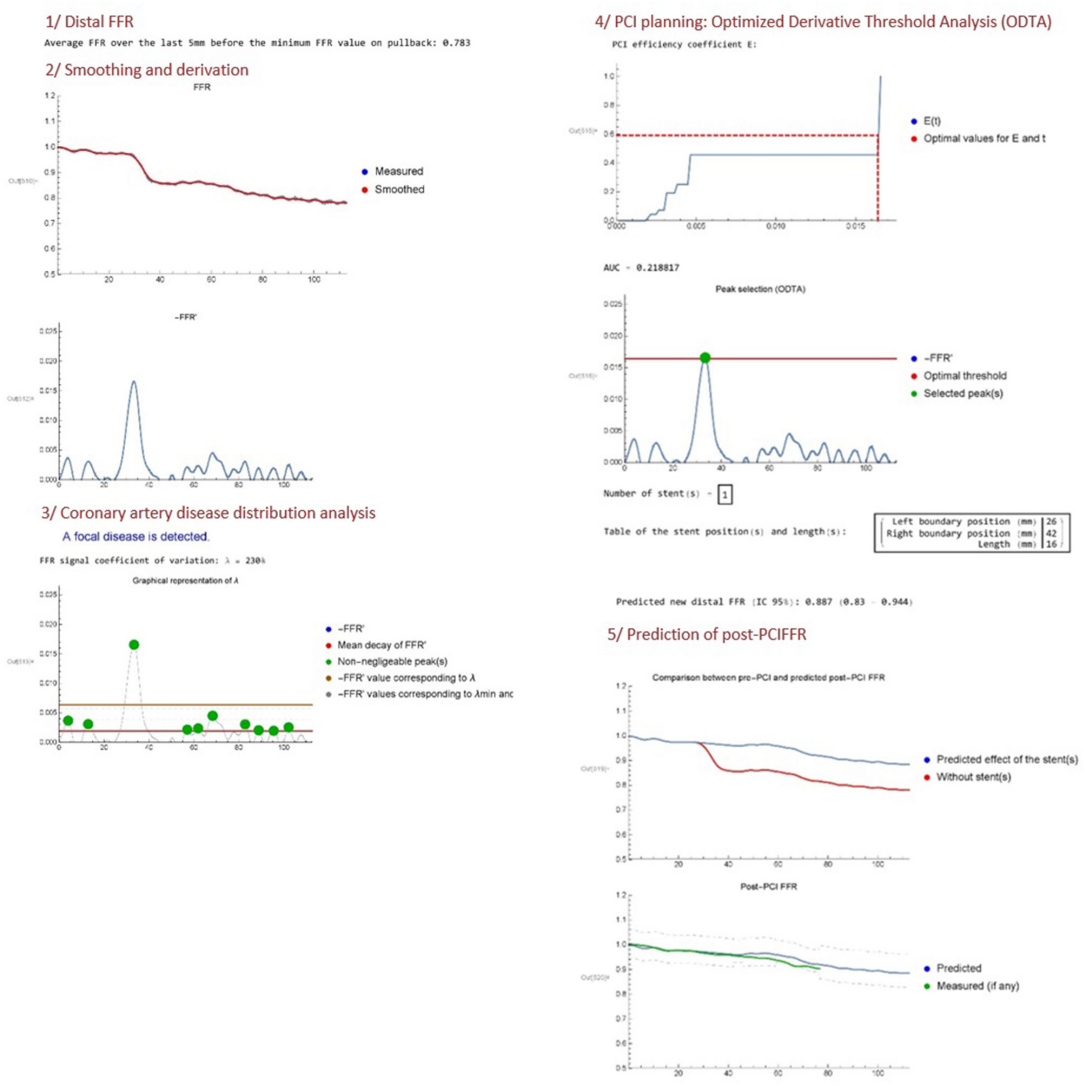
Illustrative report of VSA analysis.

Two authors (JD, JFA) developed an algorithm (Mathematica v.11.3.0) called VSA FFR-PB analyser (Copyright 2020 under GNU General Public License; I-depot evidence Benelux Office for Intellectual Property Number 123060 Date 16-04-2020) and able to perform a fully automated analysis of the FFR-PB curve in order to provide: (1) a quantitative analysis of epicardial disease distribution, and (2) a computing of ideal stent positioning in order to remove enough coronary resistance to eliminate the majority of pressure loss across the vessel. At this stage of development, the virtual stenting algorithm (VSA) is currently written in the Mathematica programming language and works offline.The input data is an excel file containing a set of values (*x, FFR*_0_(*x*)), where the pullback length *x* (mm) verifies 0 ≤ *x* ≤ *L* with *L* the total length of the pullback (mm), and *FFR*_0_(*x*) is the FFR measured in position *x*. Optionally, a data file containing the post-PCI FFR values may also be uploaded. Then, the program can be decomposed in the following steps (Figure 2).

#### Distal FFR

The distal FFR is first computed by averaging *FFR*_0_ over the last 5 mm (default setting) of the FFR recording. This mean of distal FFR over a certain length was performed in order to consider only the truly decreased distal FFR below the range of 0.8 and avoid the running of VSA in borderline cases. If the distal FFR is >0.81, the program indeed stops.

#### Smoothing and Derivation

The second step is to convert the raw FFR data *FFR*_0_(*x*) into a smoothed continuous function *FFR*(*x*) by applying a low pass filter. The goal of this filter is to get rid of higher frequency oscillations associated with breathing artifact. Indeed, adenosine infusion increases breathing amplitude which generates some cyclic oscillation in FFR measurement. Other types of non-cyclic artifacts (moving, cough) or other non-artifactual sources of pressure variations (pressure recovery phenomenon, pressure drift) were not corrected by the VSA. The cut-off frequency of the filter is *freq* = 0.004 as a default setting, but can be fine-tuned by the operator if judged necessary. Both the raw data *FFR*_0_(*x*) and the smoothed function *FFR*(*x*) are represented in the same plot. Then, the opposite of the derivative function of *FFR*(*x*), written −*FFR*′(*x*), is plotted. The minus sign is chosen as a convention in order to manipulate positive quantities (since *FFR* is generally decreasing along the vessel).

#### Coronary Artery Disease Distribution Analysis

The program then computes the mean value $\bar{-FFR^{\prime }}$ of −*FFR*′(*x*), which is simply given by

$$\begin{array}{l} \bar{-FFR^{\prime }}=\frac{FFR\left(0\right)-FFR\left(L\right)}{L}. \end{array}$$

Then, the program selects the finite set of *N* pullback lengths _(*x*_*i*_)1 ≤ *i* ≤ *N*_ associated with all the peaks (local maxima) in the −*FFR*′ function that are larger than $\bar{-FFR^{\prime }}$, using a standard Mathematica function (*FindPeaks*). Thus, we define $\bar{-FFR^{\prime }}$, the total difference quotient of *FFR* along the vessel, as a natural threshold in order to select peaks in the derivative that have a non-negligible impact on the global decrease of FFR. A modified coefficient of variation that we call “lambda” (λ) is then obtained using the following formula:

$$\begin{array}{l} \lambda =\frac{\sqrt{\frac{1}{N}\overset{N}{\underset{i=1}{\sum }}{\left(-FFR^{\prime }\left(x_{i}\right)-\bar{-FFR^{\prime }}\right)}^{2}}}{\bar{-FFR^{\prime }}}. \end{array}$$

The lambda coefficient quantifies the variation magnitude of the non-negligible peaks in −*FFR*′. A high VSA lambda FFR variation coefficient means that −*FFR*′ is characterized by large peaks arising from a focal disease, going out of the range of small variations of −*FFR*′ related to diffuse disease. We propose to use this variations of −*FFR*′ to define the distribution of coronary disease: A VSA lambda FFR variation coefficient of more than 200% is defined as a focal disease; a VSA lambda FFR variation coefficient between 100 and 200% is defined as a combined disease; whereas a VSA lambda FFR variation coefficient <100% is defined as a diffuse disease. Considering that there is no clear criteria to define. Several studies proposed focal as abrupt pattern and diffuse as gradual pattern on FFR pullback. Considering the lack of standardized definition of the physiological pattern of disease, these lambda FFR variation threshold of 100 and 200% offers a first empirical attempt to provide a non-operator dependent qualification of vessel disease distribution. The algorithm provides a plot of the VSA lambda FFR variation coefficient analysis of the −*FFR*′(*x*) curves and indicates the category of vessel disease distribution (focal/combined/diffuse).

#### PCI Planning: Optimized Derivative Threshold Analysis

The algorithm then proposes a PCI planning based on a method that we call Optimized Derivative Threshold Analysis (ODTA). The ODTA consists in the computation of a PCI efficiency coefficient written *E*(*t*) which represents the percentage of −*FFR*′ that can be relieved by the implantation of a stent for a moving threshold *t* on −*FFR*′. It is based on the following assumptions:

i The total stent length should be minimized.
ii If a stent is between positions *x*_*l*_ and *x*_*r*_ such that the pre-PCI data verifies $-FFR^{\prime }\left(x_{l}\right)=-FFR^{\prime }\left(x_{r}\right)=Z$, the post-PCI FFR verifies −*FFR*′(*x*) ≈ *Z* for all *x* between positions *x*_*l*_ and *x*_*r*_.
iii A stent generates a fixed resistance,
iv Unchanged epicardial resistance before and after the stent, and stable microvascular resistances after PCI.

We choose *Z* = −0.1/*L*, corresponding to an averaged FFR decrease of 10% along the vessel, as the defining parameter for selecting the left and right boundary positions *x*_*l*_ and *x*_*r*_ for the stent(s). Then, the PCI efficiency coefficient *E*(*t*) is defined explicitly as

$$\begin{array}{l} E\left(t\right)=1-\frac{\overset{N\left(t\right)}{\underset{i=1}{\sum }}FFR\left(x_{l,i}\right)-FFR\left(x_{r,i}\right)}{FFR\left(L\right)-FFR\left(0\right)}, \end{array}$$

with the peaks ordered by decreasing sizes, *x*_*l,i*_ and *x*_*r,i*_ the left and right boundary positions of peak *i* (respectively), and *N*(*t*) the number of peaks with maxima larger than the threshold *t*. In other words, *E*(*t*) plots the contribution of each −*FFR*′ peak higher than the moving threshold *t* to the global FFR decrease recorded into the vessel. Then, the algorithm computes the optimal threshold *t*_0_ on −*FFR*′ in order to determine which peak(s) should be removed in priority to ensure an improvement of the distal FFR at a pre-specified value of post-PCI FFR set by default at 0.87. This value was set to ensure that the inferior marge of the 95% CI of the post-PCI FFR prediction variability stays above the FFR threshold of ischemia of 0.8. This confidence interval was generated from the mean difference between the predicted post-PCI FFR and the measured post-PCI FFR, previously recorded in a subgroup of vessels (*n* = 12, data not shown). The algorithm provides then the corresponding stent length(s) and position(s) required to remove the detected peak(s) of interest. The *E*(*t*) PCI efficacy coefficient and a −*FFR*′ curve displaying the optimal threshold *t*_0_ and selected peak(s), are plotted.

#### Prediction of Post-PCI FFR

The fifth and final step includes the post-PCI FFR predictor. According to ODTA, the algorithm provides an evaluation of the expected changes in the FFR curves after the execution of the proposed treatment strategy. A first plot provides the expected changes in the FFR-PB curve and a last plot provides the confidence interval of this predicted post-PCI FFR. If available, the prediction is compared to real post-PCI data on the same plot.

VSA FFR-PB analyser (Copyright 2020 under GNU General Public License; I-depot evidence Benelux Office for Intellectual Property Number 123060 Date 16-04-2020) is accessible at https://www.wolframcloud.com/obj/jean-francois.argacha/Published/VSAFCVMrelease.nb.

### Statistics

Continuous variables with normal distribution are presented as mean ± standard deviation (SD) otherwise as median (interquartile). Categorical variables are presented as number and percentages. The χ^2^ test was used to examine the relationship between the raters, the operator, and the VSA on the classification of the coronary artery disease pattern into focal, combined or diffuse disease and PCI eligibility. A one-way ANOVA test was used to compare global stent length and groups, followed by a *post-hoc* least significant difference (LSD) test for inter-groups comparison.

The inter-rater reliability was assessed using Fleiss kappa for multiple raters. The overall and specific agreement (in percentage) were calculated based on the evaluations of the individual raters in CA/VSA setting with respect to the reference standard (the operator) and the VSA. The goal of this agreement analysis was to evaluate if the final evaluation done by raters would be at the end more in accordance with the algorithmic evaluation (VSA) or the human evaluation (operator). For this agreement analysis, only the presence of a focal disease vessel was considered as a hemodynamic eligible for PCI for the VSA.

The reclassification in raters evaluation of the three-category of vessel disease distribution and the PCI eligibility from CA to CA/FFR-PB, and from CA/FFR-PB to CA/VSA were calculated and graphically represented. The proportion of reclassification by CA/FFR-PB and CA/VSA were compared. Furthermore, the global effects of these reclassifications on the most clinically relevant categories, namely rating a vessel disease distribution as focal and considering a vessel as PCI eligible, were specifically evaluated. A “confusing effect” of CA/FFR-PB was also calculated from the number of cases where the reclassification done by CA/FFR-PB was undone by CA/VSA evaluation. A χ^2^ test was used to compare this “confusing effect” proportion arising from CA/FFR-PB with the amount of cases remaining stable throughout CA/VSA evaluation.

To assess the effect of CA/FFR-PB and CA/VSA on PCI strategy, an overall index were calculated based on following variables: change in PCI indication, change in number of lesions, change in targeted lesion position, and change in total stent length defined as more than 25% change. The overall index indicates the overall number of assessments with a change in minimum one of the four PCI strategy variables. The χ^2^test was used to compare proportion of change in PCI strategy by CA/FFR-PB and CA/VSA.

Data were analyzed using IBM® SPSS® Statistics (Version 26.0, IBM Corporation, Armonk, NY: IBM Corp.). Statistical results with *p* < 0.05 were considered statistically significant.

### Sample Size and Power Calculation

A sample size estimation was performed on based of the observations made in the second setting using the visual interpretation of angiogram and FFR pullback curves. We used the CI3Cats R-package for kappa size developed by Rotondi et al. (R software, The R Foundation for Statistical Computing) (13). The sample sizing calculation CI3Cats (kappa0 = 0.38, kappaL = 0.23, kappaU = 0.53, props = c(0.40, 0.40, 0.20), raters = 5, alpha = 0.05) indicates that a minimum of 41 subjects are required for this study of interobserver agreement.

A power calculation was also performed for the analysis of the overall agreement in vessel disease category and PCI eligibility assessment. The observed statistical power to detect significantly different agreement with operator or VSA at the α level of 5% was respectively, 100% for the vessel disease category and 56 % for the PCI eligibility. The power analysis was performed by using a on-line calculator (https://www.stat.ubc.ca/~rollin/stats/ssize/b2.html).

## Results

### Baseline Characteristics

From November 2017 to October 2019, 99 patients with 132 vessels with FFR-PB were included in a prospective coronary physiology registry. Among these, 41 vessels presented a distal FFR <0.8. After exclusion of 2 vessels because of a previous stent in the target vessel and a FFR drift > 0.02, 39 vessels (from 34 patients), were suitable for the analysis. Clinical, angiographic, and functional characteristics are summarized in Table 1. The mean pullback length was 102.9 ± 19 mm and the mean duration of adenosine infusion was 3.15 ±0.6 min. There were no adverse per procedural events associated with the motorized FFR pullback. The mean distal FFR was 0.74 ± 0.08. The mean value of the VSA lambda FFR variation coefficient was 374 ± 279%.

**Table 1:** Baseline characteristics.

**Clinical characteristics**	**34 patients**
Age, y	66.9 ± 9.6
Male, *n* (%)	24 (70.6)
BMI, kg/m^2^	28.1 ± 4.5
Hypertension, *n* (%)	26 (76.5)
Hyperlipidaemia or treatment, *n* (%)	29 (85.3)
Diabetes mellitus, *n* (%)	9 (26.5)
Active smoking, *n* (%)^*^	4 (14.3)
Prior myocardial infarction, *n* (%)	4 (11.8)
Left ventricular ejection fraction, %	53.3 ± 6.0
Creatinine, mg/dl	1.03 ± 0.4
Creatinine clearance, ml/min	74.1 ± 20.8
**Angiographic characteristics (operator assessment)**	
Vessel evaluated, *n*	39
LAD, *n* (%)	30 (76.9)
LAD - LM, *n* (%)	1 (2.6)
LCX, *n* (%)	1 (2.6)
LCX-OM, *n* (%)	2 (5.1)
RCA, *n* (%)	5 (12.8)
Diameter stenosis (%)	64.6 ± 16.1
≥ 30 and <50%, *n* (%)	8 (20.5)
≥ 50 and <70%, *n* (%)	12 (30.8)
≥ 70 and <90%, *n* (%)	19 (48.7)
Serial lesion > 50% (*n* ≥ 2), *n* (%)	13 (33.3)
**Functional characteristics**	
Pullback length, mm	102.9 ± 18.85
Distal FFR value	0.736 ± 0.09
VSA	
Distal FFR value (last 5 mm)	0.749 ± 0.08
Lambda, FFR variation coefficient	374 ± 279
E, PCI efficiency coefficient	0.54 ± 0.1

Results are displayed as n (%) unless stated otherwise.

* *total n = 28. FFR, fractional flow reserve; LAD, left anterior descending coronary artery; RCA, right coronary artery; LCX, Left circumflex coronary artery; LMT, left main; BMI, Body Mass Index; VSA, virtual stenting algorithm*.

### Operator, Raters', and VSA Assessment of Vessel Disease Distribution and PCI Eligibility

In total, 165 ratings (30 vessels reviewed by 4 raters and 9 vessels reviewed by 5 raters) were performed. Table 2 shows the vessel disease category and consecutive PCI strategy reported by the operator, raters in CA interpretation setting and VSA. Focal disease was predominantly reported by VSA (74.4 vs. 30.8 and 40% for operator and raters respectively, *p* < 0.001), diffuse disease by the operator (38.5 vs. 16.4 and 2.6% for raters and VSA respectively, *p* < 0.001), and combined disease by the raters in CA interpretation setting (43.6 vs. 30.8 and 23.1% for the operator and VSA respectively, *p* < 0.05). The raters in CA interpretation setting, VSA and the operator reported similar proportions in PCI eligibility (82.4 vs. 74.4 vs. 66.7%, *p* = 0.076). No significant difference was found in the presence of serial lesions (>1 lesion) between operator, raters and VSA (26.9 vs. 22.6 vs. 10.3 %, *p* = 0.268). The stent length differed, with longer stent length proposed by raters compared to VSA (29.42 ± 11.8 vs. 23.86 ± 10.8 mm, *p* < 0.05).

**Table 2:** Diagnostic and treatment strategies by operator, raters in CA interpretation setting and VSA.

	**Operator**	**Raters in CA setting**	**VSA**	***p*-value**
*n*	39	165	39	
Disease category, *n* (%)				
Focal	12 (30.8)	66 (40)	29 (74.4)^*^	<0.001
Combined	12 (30.8)	72 (43.6)	9 (23.1)	
Diffuse	15 (38.5)^‡^	27 (16.4)	1 (2.6)	
Vessel eligible for PCI^$^, *n* (%)	26 (66.7)	136 (82.4)	29 (74.4)	0.076
Number of lesion to be treated, *n* (%)				
One lesion	19 (73.1)	106 (77.9)	(89.7)	0.268
>1 lesion	7 (26.9)	30 (22.1)	3 (10.3)	
Stent length, mm	24.85 ± 9.77	29.42 ± 11.8^||^	23.86 ± 10.8	0.021^§^

VSA, virtual stenting algorithm.

* p < 0.05 VSA vs. 2 other categories; p < 0.05 raters vs. 2 other categories;

‡ p < 0.05 operator vs. 2 other categories;

§ One-Way ANOVA;

|| p < 0.05 LSD post-hoc analysis raters vs. VSA.

$ *Focal disease was defined as hemodynamic eligible PCI, whereas diffuse and combined as hemodynamic non-eligible for PCI*.

### Inter-Rater Reliability in Vessel Disease Assessment by CA, CA/FFR-PB, and CA/VSA

Inter-rater reliability in vessel disease assessment by CA, CA/FFR-PB, and CA/VSA were respectively, 0.32 (95% CI: 0.17–0.47), 0.38 (95% CI: 0.23–0.53), and 0.4 (95% CI: 0.25–0.55).

### Agreement in Vessel Disease Category and PCI Eligibility Assessment

**Figure 3:**
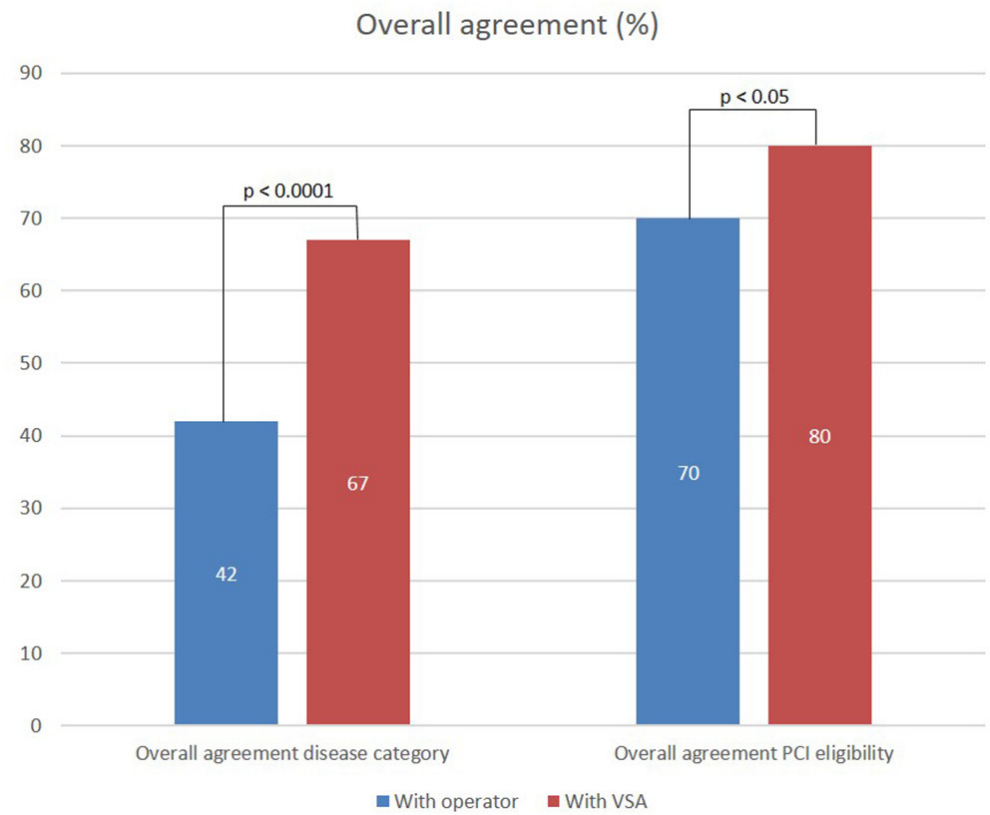
Overall agreement of ratings performed in CA/VSA setting with operator and VSA evaluation of vessel disease category and PCI eligibility.

The overall and specific agreement between the raters in CA/VSA and the operator, and the raters and the VSA on assessment of vessel disease category and PCI eligibility are presented in Figure 3. Regarding the assessment of vessel disease distribution, a higher overall agreement was found with the VSA compared to the operator (67 vs. 42%, *p* < 0.001). The agreement within the combined vessel disease category was higher with VSA than with the operator (80 vs. 16%, *p* < 0.01**)**. A trend toward a higher agreement with the operator than VSA within the focal category (76 vs. 62%, *p* = 0.09) and a higher agreement with VSA than the operator within the diffuse category (80 vs. 39%, *p* < 0.001) are observed.

Regarding the assessment of PCI eligibility, a higher overall agreement was found with VSA than with the operator (80 vs. 70%, *p* < 0.05).

### Vessel Disease Reclassification by FFR-PB Curves and VSA

**Figure 4:**
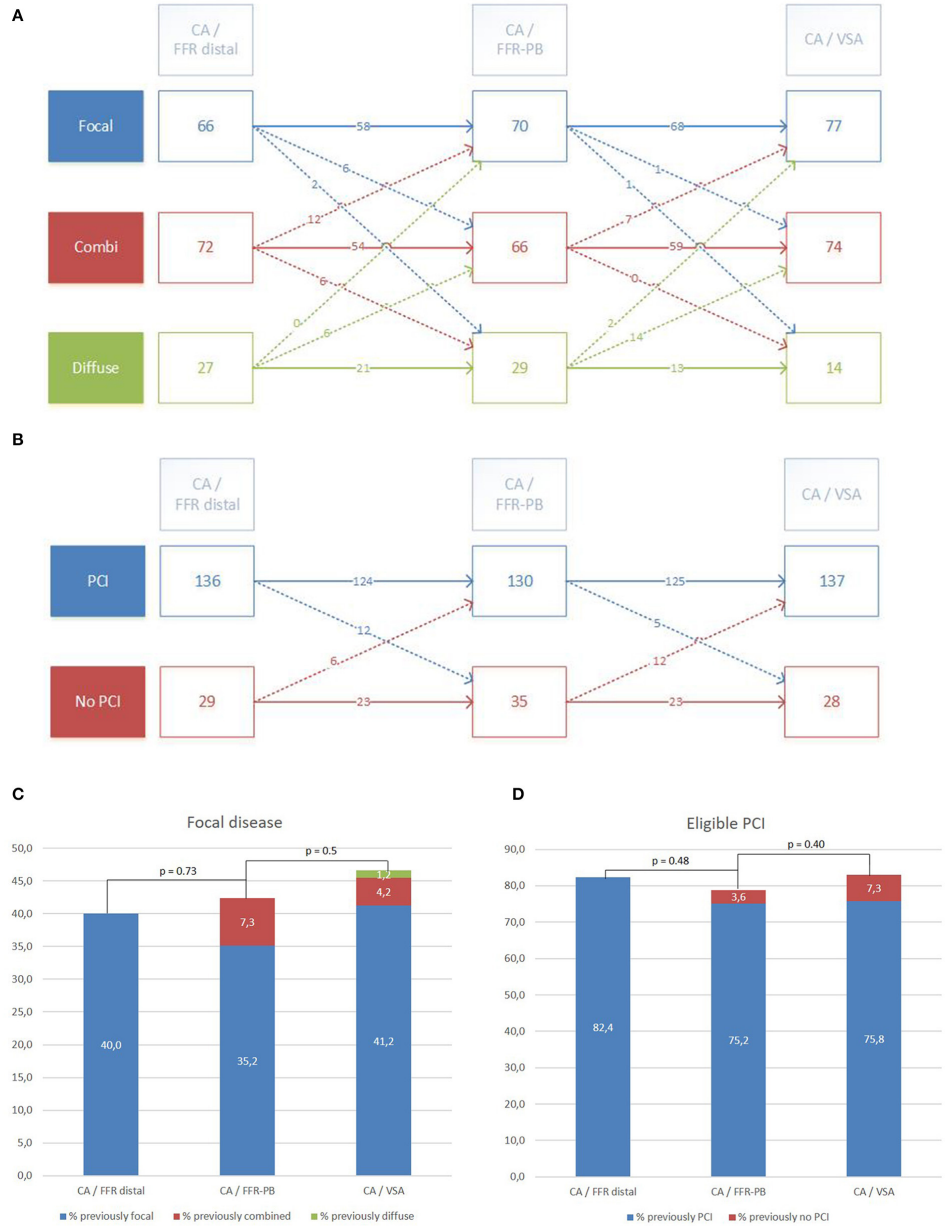
Reclassification of vessel disease category and PCI eligibility by visual interpretation (CA/FFR-PB) and VSA facilitated interpretation (CA/VSA) of FFR-PB curves. Detailed reclassification diagram of vessel disease category **(A)** and PCI eligibility **(B)**. Global trend of reclassification in focal disease category **(C)** and eligible PCI **(D)**.

Figure 4A shows the reclassification occurring in vessel disease category when raters integrate raw FFR-PB curves (CA/FFR-PB) and VSA results (CA/VSA). Compared to CA/FFR-PB, CA/VSA induces a similar rate of reclassification of vessel disease category (15.1 vs. 19.4%, *p* = 0.38). Reclassifications by CA/FFR-PB occur with a similar pattern toward focal or diffuse disease (11 vs. 8.5% respectively, *p* = 0.68). Reclassifications by CA/VSA occurred mainly toward focal than diffuse disease (14 vs. 1.2%, respectively, *p* < 0.001). As a result, CA/VSA induced more reclassification toward focal than diffuse disease compared to CA/FFR-PB (92 vs. 56.2%, *p* < 0.01).

Compared to the vessel disease category assessments without changes across the different settings, a significant proportion of reclassified assessments done by CA/FFR-PB was undone by CA/VSA (18.7 vs. 89%, *p* < 0.001). The global effect of these successive reclassifications was a subsequent non-significant trend toward an increase in the proportion of focal disease category reported by CA/FFR-PB and then by CA/VSA (p NS, Figure 4C).

### PCI Eligibility Reclassification by FFR-PB Curves and VSA

Figure 4B presents the reclassification of PCI eligibility in vessels when raters successively integrate the raw FFR curves (CA/FFR-PB) and the VSA analysis (CA/VSA). Compared to CA/FFR-PB, CA/VSA reclassified PCI eligibility in a similar proportion (10.3 vs. 11%, *p* = 1), but with a trend toward a higher reclassification as appropriate PCI (70.6 vs. 33%, *p* = 0.06).

Compared to the PCI eligibility assessments without changes across the different settings, a significant proportion of reclassified assessments done by CA/FFR-PB was undone by CA/VSA (39 vs. 93%, *p* < 0.001). No significant global effect of these successive reclassifications on the proportion of eligible PCI was observed (Figure 4D).

**Figure 5:**
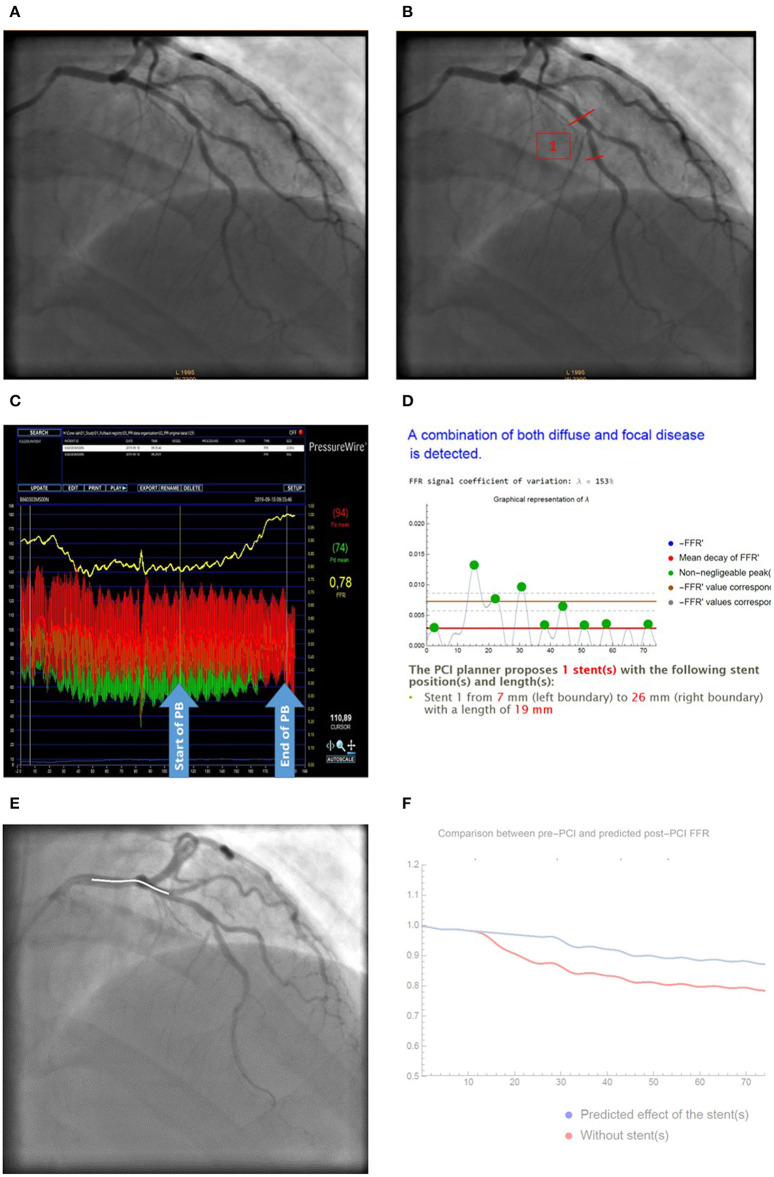
Illustrative case of change in PCI strategy by VSA analysis. **(A)** Baseline CA; **(B)** Stent positioning according to CA; **(C)** Raw FFR-PB curve; **(D)** VSA analysis reporting lambda coefficient and stent position; **(E)** Virtual stenting according to VSA; **(F)** Predicted effect of stenting on post-PCI FFR (red curves) compared to baseline FFR curves (blue curve). Based on CA **(A)**, most of raters proposed a PCI of the mid LAD-D1 bifurcation **(B)**. Facilitated interpretation of FFR-PB curves **(C)** by VSA **(D)** recommend a more proximal stenting from mm 7–26 by using a 19 mm sent over the distal LM-LAD bifurcation. FFR-PB curves **(E)**. Prediction of the effect of the stent on post-PCI FFR are presented as a curve **(F)**.

Effects of CA/FFR-PB and CA/VSA on PCI strategy are presented in Table 3. No significant change in the PCI strategy variables and its composite index was found. The proportion of assessment with at least one change in PCI strategy (overall index) did not differ between CA/FFR-PB and CA/VSA (23.6 vs. 28.5%, *p* = 0.38). An illustrative example of changes in PCI strategy by VSA is presented in Figure 5.

**Table 3:** Effect of CA/FFR-PB and CA/VSA interpretation settings on PCI strategy.

	**CA/FFR-PB**	**CA/VSA**	***p*-value**
Change in			
PCI	18 (10.9)	17 (10.3)	0.89
Number of lesions	13 (7.9)	16 (9.7)	0.70
Stent position	4 (2.4)	3 (1.8)	1
Stent length^*^	17 (10.3)	25 (15.2)	0.25
Overall index	39 (23.6)	47 (28.5)	0.38

Values are n (%);

* *Change in stent length defined as > 25% of the total stent length; Overall index: number of assessments with at least one change in PCI strategy (n = 165)*.

## Discussion

The main results of this study can be summarized as follows: we observe (1) a gain from a fair to a moderate inter-rater reliability when VSA analysis of FFR-PB curves was integrated into the standard of care evaluation of vessel disease category; (2) a higher agreement in both final raters' evaluations of vessel disease distribution and PCI eligibility with the computer algorithmic than with the operator evaluation who initially managed the case; (3) a similar rate of reclassification in vessel disease category and PCI eligibility by raw FFR-PB curve and VSA interpretations, but a decreased 'confusing effect' of the computer facilitated interpretation, (4) an increased detection of focally distributed disease by VSA, and (5) around 25% change in PCI strategy either after visual inspection or VSA assisted interpretation of the FFR-PB curves.

The lack of standardization in vessel disease evaluation and PCI eligibility evaluation may affect PCI ability to change cardiovascular outcomes, as low post-PCI FFR is frequently observed and related to poorer outcomes (14). As previously reported (15), our study confirms a low reliability of the visual interpretation of CA to define the pattern of coronary disease. When expert reviewers use either FFR-PB or VSA data, their conclusions are less variable, which should lead toward a more standardized and optimized PCI strategy. However, inter-rater reliability remains moderate likely due to the lack of consensus on what is angiographically and physiologically considered as a focal or a diffuse disease. This absence of an angiographic or physiologic gold standard definition of vessel disease distribution legitimates the development of new standards to offer a simple and non-observer dependent definition of vessel disease distribution. The hyperemic pullback pressure gradients (PPGs) and VSA and are two different approaches aiming to standardize assessment of epicardial disease distribution based on FFR pullback curves analysis. The VSA approach proposes to perform a signal to noise analysis of the magnitude of FFR derivative (FFR') signal of the entire vessel, whereas the PPG focus its analysis on the 20 mm of vessel showing the highest variation in FFR (10). The VSA physically defines a focal disease as a FFR' signal to noise ratio of more than 200%, whereas, the PPG proposes no cut-off value to consider a disease as focally of diffusely distributed. These VSA lambda coefficient thresholds still need to be validated throughout an accuracy analysis using intra-coronary imaging as gold-standard. Indeed, we notice in this proof of concept-hypothesis generating study that the focal disease category was more frequently detected by VSA analysis compared to visual inspection of the coronary angiogram. Furthermore, raters more frequently reclassify vessel disease distribution as focal disease category after the lecture of the VSA report. These findings are difficult to interpret at this stage, and may either suggest an overestimation of focal disease by the VSA, or an underestimation by the visual inspection of CA. Nevertheless, a facilitated interpretation of FFR-PB curves by a computer does not seem to generate a disproportionate rate of reclassifications. Indeed, VSA reclassifies vessel disease category in almost 15% of ratings whereas higher rates of reclassification were previously reported in studies using visual inspection of FFR and iFR coronary pressure-wire pullback (10, 16).

Sharing similar aims with the VSA, an algorithmic interpretation was also recently applied to non-hyperaemic coronary pressure wire pullbacks to facilitate and standardize their interpretations (17). A heart-team evaluation of the pressure curves, established by the consensus coming from individual rater's evaluation, was used as a gold standard. In this study, a 90% agreement between raters and algorithm evaluation was non-inferior to the agreement observed between raters and heart-team. In comparison, we report a 67% agreement between final evaluation of raters and VSA regarding vessel disease category, meaning that our raters still disagree with the suggested disease distribution by VSA in one third of cases. This lower percentage of agreement is more likely driven by our methodology, than by a less efficient computing. Indeed, raters assessment was performed in our study on 3 categories (focal, combined, diffuse), instead of 2 (focal, non-focal) in the iFR study, which intrinsically influences the reliability of the estimates. Moreover, only pressure traces were interpreted in the iFR study, whereas our study relies on a more real-life approach with a VSA report given to raters on top of the visual inspection of CA and FFR-PB curves. Regarding reclassifications, the algorithmic analysis of iFR reclassified 11% toward eligibility for PCI. Interestingly, a similar proportion of PCI eligibility reclassification was observed with the VSA but, the total number of vessels eligible for PCI was not affected in our study. This finding may suggest that algorithmic analysis of FFR by VSA is an equilibrated adjunct to clinical decision-making.

Our study highlights the disparity between angiographic and physiologic assessment on epicardial disease distribution and its possible repercussion on PCI planning. Indeed, a large proportion of PCI strategy was changed by interpretation of the FFR-PB curves. VSA can facilitate FFR pullback curve interpretation, avoiding PCI in diffuse disease with suboptimal post-PCI FFR and outcome. This concept is strengthened by the very recent demonstration that a FFR signal algorithmic analysis can be used to derive some index of disease distribution that are correlated to post-PCI FFR improvement (18). To our knowledge, the VSA remains the first computer science approach of measured FFR pullback tracings which is designed to delineate optimal stent strategy in eligible PCI cases by helping physicians to determine the best stent length and position according to vessel disease distribution. Ongoing developments of VSA are focused on real-time online application, faster acquisition of FFR-PB curves, and co-registration of stent positioning and post PCI FFR prediction with CA.

## Limitations

Two main limitations of our study need to be considered: Firstly, our sample size was initially calculated to assess the effect of VSA on the reliability of the assessment of vessel disease distribution by five raters, and to ensure enough statistical power in the comparison of raters agreement with operator and with VSA. However, the relatively small number of subjects included in this study does not allowed us to perform subgroup analysis or discern less marked changes that may have occurred in PCI strategy. Secondly, as many studies exploring new tools in the coronary physiology field, assessment of VSA was mainly performed on LAD which can limit the translation of our results to other vessels.

## Conclusion

VSA is a new automated analysis aiming to facilitate and standardize the interpretation of FFR pullback curves. Our study proves the concept that an algorithmic approach of FFR pullback curves is relevant to determine the hemodynamic eligibility of PCI, and to define a physiologically based PCI strategy. This algorithmic interpretation of FFR pullback curves improves inter-raters reliability in classifying epicardial disease as focal, combined, or diffuse and leads to reclassifications and changes in PCI strategy in a quarter of cases. Such improvement of operator reliability might ultimately optimize results of coronary stenting and avoid unnecessary procedures.

## Data Availability Statement

The raw data supporting the conclusions of this article will be made available by the authors, without undue reservation.

## Ethics Statement

The studies involving human participants were reviewed and approved by Ethics Review Committee of Universitair Ziekenhuis Brussel. The patients/participants provided their written informed consent to participate in this study. The study was performed according to the ethical guidelines of the 1975 Declaration of Helsinki and approved by the Ethics Review Committee of Universitair Ziekenhuis Brussel (B.U.N. 143201628996). Written and oral informed consent was obtained from all participating patients.

## Author Contributions

J-FA: development of the original idea and study design, operator an rater, and manuscript writing. JD: coding of the VSA and manuscript review. BV: operator, rater, and manuscript writing. DB: integration VSA stent border, coronary angiogram, and manuscript review. SL, QH, and PX: operator, rater, and manuscript review. KV: study design, data collection, statistical analysis, and manuscript writing. JM: statistical analysis and manuscript review. PS and BC: manuscript review. All authors contributed to the article and approved the submitted version.

## Conflict of Interest

QH: received research grant and speaker honoraria from Abbott Vascular. The remaining authors declare that the research was conducted in the absence of any commercial or financial relationships that could be construed as a potential conflict of interest.
